# Homozygous missense mutation (G56R) in glycosylphosphatidylinositol-anchored high-density lipoprotein-binding protein 1 (GPI-HBP1) in two siblings with fasting chylomicronemia (MIM 144650)

**DOI:** 10.1186/1476-511X-6-23

**Published:** 2007-09-20

**Authors:** Jian Wang, Robert A Hegele

**Affiliations:** 1 Schulich School of Medicine and Dentistry, University of Western Ontario and Vascular Biology Research Group, Robarts Research Institute, London, Ontario, N6A 5K8, Canada; 2 Blackburn Cardiovascular Genetics Laboratory, Robarts Research Institute, 406-100 Perth Drive, London, ON, N6A 5K8, Canada

## Abstract

**Background:**

Mice with a deleted *Gpihbp1 *gene encoding glycosylphosphatidylinositol-anchored high-density lipoprotein-binding protein 1 (GPI-HBP1) develop severe chylomicronemia. We screened the coding regions of the human homologue – *GPIHBP1 *– from the genomic DNA of 160 unrelated adults with fasting chylomicronemia and plasma triglycerides >10 mmol/L, each of whom had normal sequence of the *LPL *and *APOC2 *genes.

**Results:**

One patient with severe type 5 hyperlipoproteinemia (MIM 144650), fasting chylomicronemia and relapsing pancreatitis resistant to standard therapy was found to be homozygous for a novel *GPIHBP1 *missense variant, namely G56R. This mutation was absent from the genomes of 600 control subjects and 610 patients with hyperlipidemia. The *GPIHBP1 *G56 residue has been conserved throughout evolution and the G56R mutation was predicted to have compromised function. Her homozygous brother also had refractory chylomicronemia and relapsing pancreatitis together with early coronary heart disease. G56R heterozygotes in the family had fasting mild hypertriglyceridemia.

**Conclusion:**

Thus, a very rare *GPIHBP1 *missense mutation appears to be associated with severe hypertriglyceridemia and chylomicronemia.

## Background

Glycosylphosphatidylinositol (GPI)-anchored high-density lipoprotein (HDL)-binding protein 1 (GPIHBP1) was identified by expression cloning as a cell surface protein that bound high-density lipoprotein (HDL) [1]. Recently, mice with induced deficiency in *Gpihbp1 *showed compromised lipolysis leading to severe chylomicronemia, even on a low-fat diet [2]. GPIHBP1 appears to provide a critical platform for the binding of both lipoprotein lipase (LPL) and chylomicrons [1,2]. Since no human mutations in *GPIHBP1 *have yet been reported, we screened the genomic DNA of 160 unrelated adults with fasting chylomicronemia to search for coding sequence mutations in this gene.

## Results

### Demographics of study sample

From a tertiary referral lipid clinic, we evaluated 160 patients (33% female, 35% with diabetes) who had fasting chylomicronemia on at least one occasion. Age, body mass index, untreated fasting plasma cholesterol and triglycerides (mean ± standard deviation [SD]) were, respectively, 50.5 ± 13.8 years, 30.2 ± 4.8 kg/m^2^, 11.9 ± 6.0 mmol/L and 31.1 ± 25.0 mmol/L. All subjects consented to DNA analysis. No coding sequence mutations were found in *LPL *and *APOC2 *genes encoding, respectively, lipoprotein lipase and apolipoprotein (apo) C-II.

### Characterization of family with GPIHBP1 mutation

**Figure 1:**
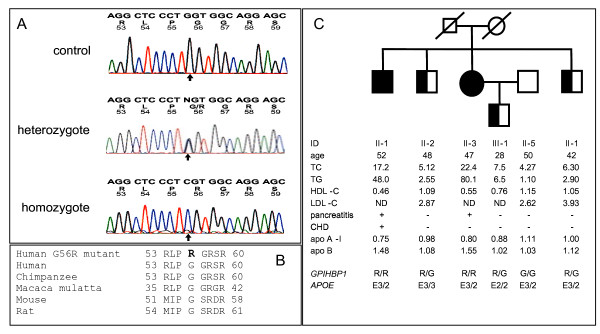
**Molecular genetic studies of *GPIHBP1***. **A) **DNA sequence analysis of *GPIHBP1 *exon 2 from genomic DNA of a normolipidemic subject (upper tracing), a G56R heterozygote (middle tracing) and the homozygote proband (lower tracing). For each tracing, normal nucleotide sequence is shown in the top line of letters, with single letter amino acid codes and codon numbers beneath. The position of the mutated nucleotide is indicated by the arrow. **B) **Evolutionary conservation of human *GPIHBP1 *G56 in primates and rodents; the single letter amino acid codes and peptide position for the homologous region from each species are shown. **C) Nuclear family of the proband with severe type V hyperlipoproteinemia and fasting chylomicronemia. **The proband (identification [ID] number II-3) and her older affected brother (ID II-1) are indicated with solid symbols. Genotype-proven heterozygotes are shown with half-solid symbols. Patient age and plasma concentrations of total cholesterol (TC), triglyceride (TG), high-density lipoprotein cholesterol (HDL-C) and low-density lipoprotein cholesterol (LDL-C), all in millimoles per litre (mM), and of apolipoprotein (apo) A-I and B, in grams per litre (g/L), are shown. ND means the value could not be determined. A documented history of hospitalization for pancreatitis and coronary heart disease (CHD) are shown. Genotypes of the *GBPHBP1 *exon 2 G56R mutation and of common *APOE *isoforms are shown.

Only one rare coding sequence variant in *GPIHBP1 *was found among the 160 screened patients, namely G56R (Figure 1A). This missense mutation was absent from the genomes of 600 normolipidemic Caucasian control subjects and 610 Caucasian patients with hyperlipidemia. The mutated amino acid residue was evolutionarily conserved (Figure 1B) and analysis with the PolyPhen algorithm [3] indicated that the mutation was probably damaging.

The proband, a homozygote for *GPIHBP1 *G56R, had relapsing pancreatitis beginning at age 22 and was documented on numerous occasions to refractory fasting chylomicronemia, even with fat restriction. She had no thyroid, renal or hepatic disease and was not diabetic. She was not obese and consumed no alcohol. Her older brother had a similar biochemical profile, with a history of relapsing pancreatitis requiring hospitalization, refractory to medical treatment since age 25. At age 45 he required 3-vessel coronary artery bypass graft surgery for unstable angina symptoms that began at age 44 (Figure 1C).

Both patients had normal activities of lipoprotein and hepatic lipases in post-heparin plasma, indicating that *ex vivo *lipolytic activity was not compromised. Both parents were long-deceased. Although consanguinity was not documented, it was possible since both parents were born in the same village. Three heterozygotes in this pedigree each had plasma triglyceride concentration in the top 5^th ^percentile for age and sex, but no history of pancreatic or cardiovascular disease. Further, the proband's untreated son had combined hyperlipidemia, with approximately equimolar elevations of plasma total cholesterol and triglycerides, which together with an *APOE *E2/E2 genotype were highly suggestive of type 3 hyperlipoproteinemia (dysbetalipoproteinemia). Both patients have had a variable response to oral fibrate therapy, with a somewhat better response to restriction of fat intake to 20% of calories and to omega-3 fatty acids, although long term compliance has been an issue. Plasma triglyceride concentration was never <10 mmol/L in either patient over since their diagnosis.

## Discussion

The recent characterization of a focal role for Gpihbp1 in murine triglyceride metabolism was a significant development in the lipoprotein field [2]. Our genetic findings suggest that GPIHBP1 might also have an important role in human triglyceride metabolism, albeit only one missense mutation in *GPIHBP1 *– G56R – was found among 160 patients with an analogous phenotype to mice with a deleted *Gpihbp1 *gene, namely severe type 5 hyperlipoproteinemia, fasting chylomicronemia and a normal coding sequence of *LPL *and *APOC2 *genes. The G56R mutation altered a highly conserved residue and functional compromise was predicted. The mutation was associated in homozygotes with severe, refractory hypertriglyceridemia. In heterozygotes, the mutation was associated with mild to moderate hypertriglyceridemia, and very likely with type 3 hyperlipoproteinemia in a subject who was also homozygous for *APOE *E2/E2. Furthermore, the G56R mutation was absent from large numbers of normolipidemic control subjects and also from a large number of patients referred to a tertiary clinic for management of a wide range of hyperlipidemia phenotypes.

## Conclusion

The association of this coding sequence variant with severe type 5 hyperlipoproteinemia with chylomicronemia, its absence from normolipidemic subjects together with other evidence for its likely pathogenicity appears to link the *GPIHBP1 *gene with severe human metabolic phenotypes. The fact that this was the only mutation from hundreds of screened subjects with an appropriate disease phenotype indicates that mutations in this gene are not likely to be a major cause of severe hypertriglyceridemia.

## Methods

### Genomic DNA analysis

The exons and intron-exon boundaries of *GPIHBP1 *were amplified and bi-directionally sequenced using the primers and conditions in Table 1. All coding sequence mutations were confirmed by a second sequencing reaction performed on another day. The frequency of the *GPIHBP1 *exon 2 G56R coding single nucleotide variant in 600 normolipidemic Caucasian control subjects and 610 Caucasian patients with hyperlipidemia from a tertiary referral clinic was determined using genomic DNA amplification with primers 5'-ATG CTT GCC CAG AGC AGG TGT C and 5'-GCC TGC TGG CTT CCA TCA CAC. The 282 bp product was digested with restriction enzyme *Bst*NI (New England Biolabs, Mississauga, ON) and fragments were resolved on 2% agarose gels. The fragments of the wild-type G56 allele were 114, 67, 59 and 42 bp in size, while the fragments of the R56 allele were 173, 67 and 42 bp in size. In family members, measurements of fasting plasma lipoproteins and apolipoproteins, *APOE *genotype analysis and, in the two homozygotes, assessment of lipase activities in post-heparin plasma were performed as described [4-6].

**Table 1:** Amplification primers for *GPIHBP1 *coding sequence

exon	primer sequence	annealing temperature (°C)	fragment size (base pairs)
1	5'-CCT TCA TCC CAC TTA CCG CAG C	60	276
	5'-GCC AGC TTC CAT CCA TGC TGC		
2	5'-ATG CTT GCC CAG AGC AGG TGT C	60	282
	5'-GCC TGC TGG CTT CCA TCA CAC		
3	5'-AGG CTA GGC TTT GGG AGC ACA G	60	307
	5'-GTCTCTGAGGTGGCTCTGCAG		
4	5'-CTG CAG AGC CAC CTC AGA GAC	60	447
	5'-CTG GAT CGC CCA AGA CAC TCC		

## Competing interests

The author(s) declare that they have no competing interests.

## Authors' contributions

JW carried out all molecular analysis and participated in manuscript writing. RAH conceived of the study, participated in its design, analysis, interpretation and manuscript preparation. Both authors approved the final version of the manuscript.
